# Factors influencing post discharge activities of daily living in patients receiving rehabilitation in acute care hospital

**DOI:** 10.1038/s41598-025-19186-1

**Published:** 2025-09-29

**Authors:** Hirotomo Shibahashi, Yuya Takakubo, Miyuki Murakawa, Michiaki Takagi, Masayasu Murakami

**Affiliations:** 1 Department of Rehabilitation, School of Health Sciences, Tokyo University of Technology, Tokyo, 144-0051 Japan; 2 Department of Orthopedic Surgery, Yamagata University Faculty of Medicine, Yamagata Prefecture, Yamagata, 990-9585 Japan; 3 Rehabilitation Unit, Yamagata University Hospital, Yamagata Prefecture, Yamagata, 990- 9585 Japan; 4 Department of Health Policy Science, Graduate School of Medical Science, Yamagata University, Yamagata prefecture, Yamagata, 990-9585 Japan

**Keywords:** Acute care hospital, Rehabilitation, Instrumental activities of daily living, Health care, Medical research, Risk factors

## Abstract

Post-discharge functional outcomes in patients undergoing acute rehabilitation—especially regarding activities of daily living (ADL) and instrumental activities of daily living (IADL)—remain inadequately understood. The combined effects of demographic factors and in-hospital variables on ADL and IADL status at discharge has not been clearly defined. This study aimed to clarify these associations to support effective discharge planning and post-discharge care strategies. We retrospectively analyzed 309 adult patients who received rehabilitation in an acute care hospital in Japan between April and September 2016. Patients who underwent only assessments, were pediatric, deceased, or did not receive rehabilitation were excluded. Demographic data and Barthel Index scores at rehabilitation initiation and discharge were extracted. Post-discharge ADL and IADL status were assessed approximately 1.5–2 years later using a questionnaire based on the Frenchay Activities Index (FAI). Among the 309 patients, 57 were transferred, 249 discharged home, and 3 institutionalized. ADL and IADL scores were significantly higher in the home discharge group. Higher ADL scores at discharge and post-discharge were independently associated with higher FAI scores, underscoring the importance of ADL independence for post-discharge IADL recovery. Addressing ADL limitations and optimizing rehabilitation may enhance IADL outcomes and support independent living after discharge.

## Introduction

Acute rehabilitation plays a pivotal role in the recovery trajectory of patients, particularly in maintaining physical function, promoting independence in daily activities, and preventing systemic risks such as disuse syndrome. Its ultimate aim is to ensure a safe discharge, either to the patient’s home or a suitable recovery facility.

Demographic variables, such as age, sex, and living conditions, significantly influence discharge outcomes and functional recovery. For instance, older age is associated with reduced activities of daily living (ADL) independence and an increased likelihood of institutionalization or transfer to another facility^1,2^. Measures of ADL and instrumental activities of daily living (IADL) are particularly effective in predicting hospital outcomes, often surpassing traditional medical indices in forecasting discharge destinations and recovery^3^. Furthermore, pre-hospital functional decline and in-hospital recovery are critical determinants of post-discharge outcomes, including institutionalization and mortality^1,2^. Despite these findings, the combined impact of demographic factors, ADL/IADL recovery, and discharge destinations in acute care settings remains insufficiently explored.

Understanding these relationships is crucial for optimizing discharge planning and post-discharge care. Although acute rehabilitation strategies aim to promote functional recovery and facilitate smooth transitions, limited evidence exists regarding patients’ ADL and IADL status following discharge from acute care hospitals.

Validated scales, such as the Functional Independence Measure (FIM)^4^ and Barthel Index (BI)^5^, are widely used to evaluate ADLs and predict discharge outcomes in acute rehabilitation. For IADLs, tools such as the Frenchay Activities Index (FAI)^6–8^ are particularly useful in assessing post-discharge functional status in older adults. However, there is a paucity of evidence on how demographic factors interact with ADL/IADL recovery to influence discharge outcomes.

Patients in acute care hospitals are typically classified into two groups: those who are sufficiently independent or supported to return home, referred to as the home discharge group, and those who require additional medical care or rehabilitation, known as the transfer group. Previous studies have highlighted associations between hospitalization factors, such as rehabilitation duration and length of stay, and functional outcomes^9,10^. Nonetheless, the combined influence of in-hospital factors and patient demographics on post-discharge ADL/IADL status requires further investigation.

To address these knowledge gaps, the present study aimed to examine the associations between demographic factors, in-hospital variables, and ADL/IADL status at discharge among patients receiving acute rehabilitation. By clarifying these relationships, this study seeks to provide evidence to support more effective rehabilitation practices and discharge planning in acute care settings.

## Methods

### Study design and sample

This was a retrospective observational study conducted at the Yamagata University Hospital, Japan. Between April 1 and September 30, 2016, a total of 759 patients were prescribed physiotherapy or occupational therapy by physicians. We excluded patients who underwent only functional assessments without intervention, pediatric patients, patients who died before the survey period, and patients who were prescribed but did not receive rehabilitation. After applying these criteria, 654 patients were deemed eligible for analysis. Data were extracted from electronic medical records between February 7 and April 30, 2018. This timeframe allowed for the completion of ethical approvals and dataset preparation, while enabling the evaluation of post-discharge outcomes approximately 1.5 to 2 years after discharge, capturing both short- and longer-term functional status.

## Data collection

This study was approved by the Institutional Review Board of Yamagata University Faculty of Medicine (approval no. 497). Given the large sample size and challenges in direct participant contact, an opt-out consent method was used. Study details were disclosed on the Institutional Ethics Committee website for 1 month, during which patients could decline participation. We acknowledge that this opt-out approach may have introduced potential selection bias.

Data extracted from electronic medical records included age, sex, days of rehabilitation, length of hospital stay, discharge destination (home or transfer), primary department, and BI scores at rehabilitation initiation and discharge. A follow-up postal survey was conducted between June 4 and June 30, 2018, targeting patients who had been discharged, to assess post-discharge functional status. The questionnaire described the study’s objectives, data confidentiality measures, and voluntary participation. It explained that returning the completed questionnaire would be considered informed consent, and that withdrawal at any time would not result in any disadvantage. Returned questionnaires were anonymized and assigned unique study IDs for analysis, while data from those who declined participation were excluded.

The self-administered questionnaire assessed ADL and IADL at the time of survey administration (approximately 1.5–2 years post-discharge). We developed a 32-item ADL scale, expanding upon the motor components of the FIM and incorporating culturally relevant daily activities. Each item was rated on a 7-point scale (1 = “unable to perform” to 7 = “independent”), resulting in a total score range of 32–224, with higher scores indicating greater independence. Domains assessed included personal hygiene, eating and drinking, dressing, mobility, and toileting. This scale was developed to address the limitations of existing ADL measures by providing a finer gradation of independence levels and including culturally specific activities, enhancing the accuracy and relevance of ADL assessments in diverse populations. IADL were assessed using the FAI, a validated and reliable instrument comprising 15 items scored on a 0–3 scale (total score 0–45), with higher scores indicating greater functional ability^11^. The FAI was selected over the Lowton IADL scale^12^ due to its broader scope and detailed itemization, including activities such as laundry and meal preparation, which are increasingly relevant across genders.

Post-discharge rehabilitation was not assessed due to practical limitations, and its potential impact on ADL and IADL recovery is acknowledged as a limitation of this study. We focused on in-hospital factors that are more directly modifiable within the acute care setting.

**Fig. 1:**
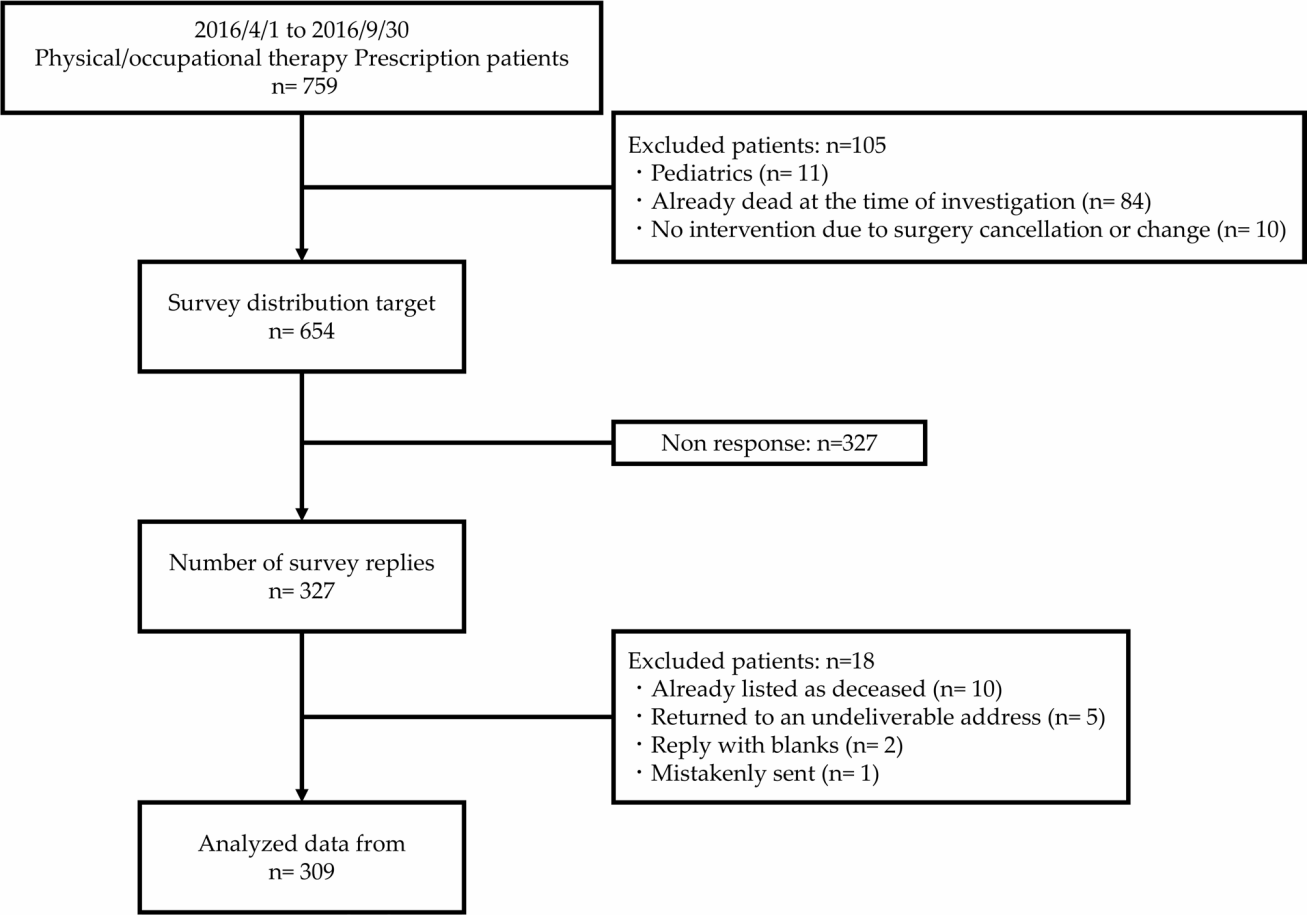
Flowchart illustrating the selection processe of study participants for inclusion in the analysis.

Of the 654 patients surveyed, 327 responses (50.0%) were returned. After excluding responses due to patient death (*n* = 10), unknown addresses (*n* = 5), incomplete responses (*n* = 2), and erroneous mailing (*n* = 1), a total of 309 valid responses were included in the final analysis, yielding an effective response rate of 47.2% (Fig. 1). Potential selection and non-response biases were considered when interpreting the results.

### Statistical analysis

The internal consistency of the newly developed ADL scale was assessed using Cronbach’s alpha, with a threshold value of ≥ 0.70 considered acceptable for reliability. Descriptive statistics were calculated for all variables. Group comparisons between the home discharge and transfer groups were performed using Fisher’s exact test for categorical variables and either Welch’s t-test or the Mann–Whitney U-test for continuous variables, depending on distribution characteristics.

To identify factors influencing the FAI score, univariate linear regression analyses were performed for each group, followed by multivariate linear regression analyses with the FAI score as the dependent variable. Independent variables included sex, age, days of rehabilitation, length of hospital stay, BI scores at the start of rehabilitation and at discharge, and scores from the the newly developed ADL assessment scale. Multicollinearity was assessed using variance inflation factors, with values > 10 indicating potential multicollinearity. Missing data, which accounted for < 10% of the dataset, were addressed using multiple imputation with chained equations to minimize bias and enhance robustness^13,14^. Fifty imputed datasets were generated and pooled for analysis under the assumption that data were missing at random. Sensitivity analyses were not performed due to the limited scope of the study; however, the potential impact of imputation on the results is acknowledged as a limitation.

Effect sizes were calculated to complement *p*-values and to quantify the magnitude of observed differences. Cramér’s V was computed for categorical variables to assess the strength of associations, interpreted as small (0.1), medium (0.3), and large (0.5) according to Cohen’s guidelines. For continuous variables, Cohen’s d was calculated where applicable, with values of 0.2, 0.5, and 0.8 interpreted as small, medium, and large effect sizes, respectively. For non-parametric comparisons, the rank-biserial correlation (r) was calculated, with thresholds for interpretation consistent with Cohen’s guidelines. In the multivariate regression analyses, standardized regression coefficients (β) were used as indicators of effect size to facilitate interpretation of the relative influence of each variable on the FAI score.

All statistical analyses were conducted using R (version 4.2.1, R Foundation for Statistical Computing, Vienna, Austria) and EZR (version 1.56, Saitama Medical Center, Jichi Medical University, Saitama, Japan)^15^. A two-tailed *p*-value of < 0.05 was considered statistically significant.

## Results

The basic attributes and clinical outcomes of the participants are shown in Table 1, categorized into two groups: those transferred to another facility (transfer group, *n* = 57) and those discharged home (home discharge group, *n* = 249).

**Table 1:** Comparison of the transfer and home discharge groups for each item.

		Transfer group (*n* = 57)	Home discharge group (*n* = 249)	*p*-value	Effect size
Sex	Male (persons) (%)	35 (11.4)	128 (41.8)	0.20*	V = 0.07
	Female (persons) (%)	22 (7.2)	121 (39.5)		
Age (years) (mean ± SD)		70.5 ± 15.5	67.7 ± 15.9	0.22†	d = 0.18
Length of hospital stay (days) (IQR)		29 (23–56)	24 (13–36)	< 0.01‡	*r* = 0.23
Days of rehabilitation during hospitalization (days) (IQR)		16 (10–32)	11 (5–17)	< 0.01‡	*r* = 0.22
BI at the start of rehabilitation (points) (IQR)		25 (0–55)	85 (50–100)	< 0.01‡	*r* = 0.36
BI at discharge (points) (IQR)		70 (45–85)	100 (95–100)	< 0.01‡	*r* = 0.65
Total score on the newly developed ADL assessment scale (points) (IQR)		199 (132–222)	224 (210–224)	< 0.01‡	*r* = 0.29
	0–100 (persons) (%)	12 (21.0)	17 (6.8)	< 0.01*	V = 0.21
	101–200 (persons) (%)	17 (29.8)	27 (10.8)		
	201– (persons) (%)	28 (49.1)	205 (82.3)		
FAI (points)(IQR)		12 (2–21)	21 (10–31)	< 0.01‡	*r* = 0.22
By department (persons) (%)					
	Orthopedic surgery	20 (35.1)	89 (35.7)	< 0.01*	V = 0.32
	Neurosurgery	11 (19.3)	22 (8.8)		
	Cardiovascular surgery	7 (12.7)	36 (14.5)		
	Respiratory surgery	1 (1.8)	37 (14.9)		
	Cardiovascular medicine	6 (10.5)	25 (10.0)		
	Neurology and hematology	4(7.0)	14 (5.6)		
	Respiratory Medicine	3 (5.3)	9 (3.6)		
	Urology	4 (7.0)	0		
	Gastroenterology	1 (1.8)	6 (2.4)		
	Breast Cancer and Gastrointestinal Surgery	0	3 (1.2)		
	Otorhinolaryngology	0	1 (0.4)		
	Oncology	0	1 (0.4)		
	Dermatology	0	4 (1.6)		
	Collagen Disease Medicine	0	2 (0.8)		

SD, standard deviation; IQR, interquartile range; BI, Barthel index; ADL, activities of daily living; FAI, Frenchay activities index; V, Cramér’s V; d, Cohen’s d; r, Rank-biserial correlation.

* Fisher’s exact test; †, unpaired t-test; ‡, Mann–Whitney U test.

Significant differences were observed between the groups in terms of duration of hospital stay (*p* < 0.01, *r* = 0.23), days of rehabilitation during hospitalization (*p* < 0.01, *r* = 0.22), BI at the start of rehabilitation (*p* < 0.01, *r* = 0.36) and at discharge (*p* < 0.01, *r* = 0.65), the total score on the newly developed ADL assessment scale (*p* < 0.01, *r* = 0.29), and the FAI score after discharge (*p* < 0.01, *r* = 0.22). Additionally, the distribution of patients across hospital departments differed significantly between groups (*p* < 0.01, V = 0.32), with orthopedics being the most common department, followed by neurosurgery and cardiovascular surgery.

The Cronbach’s alpha coefficient for the newly developed ADL assessment was 0.993, indicating high internal consistency and reliability. Effect sizes were interpreted according to standard thresholds, indicating small to large magnitudes depending on the variable, thereby complementing the statistical significance and providing context for clinical relevance.

Univariate regression analysis demonstrated that, within the transfer group, higher BI scores at discharge (β = 0.27, 95% confidence interval (CI): 0.01–0.53, *p* < 0.05) and higher total scores on the newly developed ADL assessment scale (β = 0.60, 95% CI: 0.39–0.82, *p* < 0.01) were significantly associated with higher FAI scores after discharge. The positive β values indicated that greater functional independence at discharge and higher ADL performance levels were linked to better post-discharge IADL. These standardized regression coefficients serve as effect size measures, providing additional context to the statistical significance and supporting the clinical relevance of these associations (Table 2).

**Table 2:** Univariate regression analysis of factors influencing the Frenchay activities index for the transfer group.

Variable	β	95% CI	*p*-value
Sex (female)	−0.02	−0.29-0.25	0.90
Age (years)	−0.25	−0.51-0.01	0.06
Days of rehabilitation during hospitalization (days)	−0.18	−0.44-0.08	0.17
Length of hospital stay (days)	−0.20	−0.46-0.07	0.14
BI at the start of rehabilitation (points)	0.04	−0.23-0.31	0.78
BI at discharge (points)	0.27	0.01-0.53	< 0.05
Total score on the newly developed ADL assessment scale (points)	0.60	0.39-0.82	< 0.01

β, standardized partial regression coefficient; CI, confidence interval; BI, Barthel index; ADL, activities of daily living.

Univariate regression analysis in the home discharge group demonstrated that female sex (β = 0.14, 95% CI: 0.02–0.26, *p* < 0.05), higher age (β = −0.24, 95% CI: −0.37 – −0.12, *p* < 0.01), higher BI scores at discharge (β = 0.33, 95% CI: 0.21–0.45, *p* < 0.01), and higher total scores on the newly developed ADL assessment scale (β = 0.45, 95% CI: 0.34–0.56, *p* < 0.01) were significantly associated with FAI scores. The positive β coefficients indicate that greater independence in ADL and higher BI scores at discharge were associated with better IADL performance post-discharge, whereas the negative β for age suggests that older age was associated with lower FAI scores. These standardized regression coefficients serve as effect size measures, complementing statistical significance and supporting the clinical relevance of the observed associations (Table 3).

**Table 3:** Univariate regression analysis of factors influencing the Frenchay activities index for the home discharge group.

Variable	β	95% CI	*p* value
Sex (female)	0.14	0.02-0.26	< 0.05
Age (years)	−0.24	−0.37-−0.12	< 0.01
Days of rehabilitation during hospitalization (days)	0.03	−0.12-0.15	0.68
Length of hospital stay (days)	−0.01	−0.14-0.11	0.87
BI at the start of rehabilitation (points)	0.11	−0.02-0.23	0.10
BI at discharge (points)	0.33	0.21-0.45	< 0.01
Total score on the newly developed ADL assessment scale (points)	0.45	0.34-0.56	< 0.01

β, standardized partial regression coefficient; CI, confidence interval; BI, Barthel index;

ADL, activities of daily living.

Multiple regression analysis with FAI as the dependent variable showed that in the transfer group, older age (β = −0.31, 95% CI: −0.55 – −0.08, *p* < 0.05) and higher total scores on the newly developed ADL assessment scale (β = 0.57, 95% CI: 0.37–0.78, *p* < 0.01) were significant predictors. In the home discharge group, female sex (β = 0.14, 95% CI: 0.04–0.25, *p* < 0.01), older age (β = −0.15, 95% CI: −0.26 – −0.04, *p* < 0.01), more days of rehabilitation during hospitalization (β = 0.18, 95% CI: 0.005–0.36, *p* < 0.05), and higher ADL assessment scores (β = 0.49, 95% CI: 0.36–0.61, *p* < 0.01) were significantly associated with FAI scores (Tables 4 and 5).

**Table 4:** Multiple regression analysis for the transfer group.

Variable	β	95% CI	*p* value
Sex (female)	0.08	−0.14-0.29	0.47
Age (years)	−0.31	−0.55-−0.08	< 0.05
Days of rehabilitation during hospitalization (days)	0.08	−0.64-0.80	0.83
Length of hospital stay (days)	−0.18	−0.89-0.54	0.62
BI at the start of rehabilitation (points)	−0.04	−0.26-0.18	0.71
BI at discharge (points)	0.14	−0.08-0.37	0.21
Total score on the newly developed ADL assessment scale (points)	0.57	0.37-0.78	< 0.01

β, standardized partial regression coefficient; CI, confidence interval; BI, Barthel index; ADL, activities of daily living.

**Table 5:** Multiple regression analysis for the discharge home group.

Variable	β	95% CI	*p* value
Sex (female)	0.14	0.04-0.25	< 0.01
Age (years)	−0.15	−0.26-−0.04	< 0.01
Days of rehabilitation during hospitalization (days)	0.18	0.005-0.36	< 0.05
Length of hospital stay (days)	−0.15	−0.33-0.02	0.09
BI at the start of rehabilitation (points)	0.02	−0.09-0.14	0.69
BI at discharge (points)	0.12	−0.004-0.24	0.06
Total score on the newly developed ADL assessment scale (points)	0.49	0.36-0.61	< 0.01

β, standardized partial regression coefficient; CI, confidence interval; BI, Barthel index; ADL, activities of daily living.

## Discussion

Patients discharged from an acute care hospital were categorized by outcome, and factors during hospitalization and their association with ADL and IADL abilities both during and after discharge were analyzed. Our results showed that ADL and IADL scores were significantly higher in the home discharge group than in the transfer group. Furthermore, ADL status at discharge and post-discharge significantly influenced the FAI in both groups. These findings indicate a strong relationship between the level of independence in ADLs and IADLs during and after hospitalization. Past reports have shown the effectiveness of post-discharge rehabilitation^16–20^, and notably, follow-up interventions tailored to patients’ individual needs and ADL/IADL levels appear crucial, particularly for those with low ADL independence upon discharge. Recent systematic reviews also support the effectiveness of early supported discharge interventions in reducing length of hospital stay among older adults, while highlighting the need for further high-quality research to clarify their impact on functional outcomes^21,22^. In this study, IADL data were obtained following discharge from an acute care hospital. Although there were differences in diseases and conditions among the groups, we believe that this differentiation, along with the observed correlation between post-discharge data and in-hospital data, contributes significantly to the novelty of our findings.

Previous research in Japan has indicated an association between ADL decline and subsequent IADL decline^23^. Furthermore, low ADL ability is presumed to indicate lower physical or cognitive function. In the past, sarcopenia and gait speed have been reported to be associated with lower IADL^23–27^. Furthermore, cognitive function, assessed in terms of the Mini-Mental State Examination score, has been reported to be a predictor of IADL^23,28^, and a decline in intellectual activities, such as reading books and newspapers, is also a predictor of IADL decline^29–31^. Based on these observations, we believe that the role of rehabilitation in acute care hospitals must include interventions targeting post-discharge IADL function. This approach, alongside efforts to prevent secondary complications such as disuse syndrome and associated declines in physical and cognitive functions, can maximize quality of life improvements for patients.

Additionally, age has been cited as one of the factors that decrease IADL^23,31^. The average age of the participants in this study was 65 years or older, and in the multiple regression analysis of the home discharge group, the age variable showed a significant difference, indicating a decrease in the FAI. In the home discharge group, it is necessary to take measures to address IADL decline risks in older patients.

Furthermore, the significant differences in patient distribution across hospital departments may reflect variations in underlying disease characteristics, comorbidities, and functional recovery potential associated with each department. These factors may influence both discharge destinations and post-discharge IADL outcomes, highlighting the need for department-specific considerations in discharge planning and follow-up rehabilitation strategies.

This study had some limitations. First, it relies on self-reported data from patients, which may introduce bias or inaccuracies and affect the validity of the findings. Furthermore, the ADL questionnaire used in this study was developed based on the FIM, a well-established evaluation method conducted by trained personnel. However, its adaptation into a questionnaire format has not been fully validated. Although a high Cronbach’s alpha coefficient demonstrates good internal consistency, further research is required to evaluate inter-rater and intra-rater reliability, as well as construct and criterion-related validity. These limitations may have influenced the results of the study. To enhance the reliability and applicability of the findings, future studies should validate the questionnaire in larger and more diverse populations. Second, a questionnaire assessing ADL and IADL was distributed to patients who received rehabilitation during a specified period. However, the timing of questionnaire completion varied owing to differences in the time elapsed since discharge. This variation is a limitation, as disparities in post-discharge duration may have influenced patients’ responses. Furthermore, the questionnaire did not collect detailed information on the number, content, or duration of rehabilitation interventions after discharge. These factors could substantially impact patient outcomes and should be addressed in future studies. To improve the reliability of findings, future research should standardize post-discharge assessment timing and include comprehensive data on post-discharge rehabilitation interventions. Third, this study did not account for the effects of medications used by participants. Since medication use could affect IADLs, future studies should include this factor in their analyses. Fourth, the participants’ environment is an important factor affecting IADLs; however, it was difficult to accurately investigate this in this study. Future research should analyze environmental factors. Fifth, the study employed an opt-out consent method due to the logistical challenges associated with enrolling a large sample, which may have introduced potential selection bias. Patients who did not access the institutional website or did not actively decline participation might have been inadvertently included, potentially affecting sample representativeness. However, the demographic and clinical characteristics of the included patients were comparable to the general patient population at the hospital, suggesting that the impact of this bias may be limited. Sixth, although the response rate was 50%, the study did not analyze the reasons for non-response or potential differences between respondents and non-respondents. This introduces the possibility of non-response bias, which may limit the generalizability of the findings. Future studies should examine the characteristics of non-respondents and the reasons for non-response to better understand and address this potential bias. Finally, this observational, nonrandomized study was conducted in a single acute care hospital, which limits the generalizability of our findings. The relatively small sample size limited our ability to fully adjust for influential factors, such as cognitive function, health-related quality of life, environment and social participation, inconsistent rehabilitation, surgery, and detailed general conditions that may affect patients’ IADLs. Furthermore, this study did not evaluate ADL or IADL at the time of admission, making it difficult to determine the impact of hospitalization on functional outcomes. Additionally, differences in discharge destinations, such as returning home or transitioning to another facility, may have influenced the results but were not thoroughly analyzed. This study also did not analyze BI score changes in detail for the group that was not discharged to home. It is possible that the BI scores in this group were already low at admission, minimizing the impact of hospitalization on their ADL. Future studies should investigate the role of hospitalization in functional improvement for this subgroup. Moreover, although our regression models identified significant predictors of post-discharge IADL performance, the potential influence of unmeasured confounders such as baseline cognitive status, comorbidities, and social support may have impacted these associations. The relatively modest sample size may have limited the precision of the estimated effects, and the models’ predictive strength should be interpreted with caution. This study applied multiple imputation methods to address missing data, although the proportion of missing values was less than 10%. While this approach reduces bias associated with missing data, it may have influenced the findings of the single and multiple regression analyses examining factors affecting the FAI. Additionally, as sensitivity analyses were not conducted to compare imputed datasets with complete-case analyses, the potential impact of multiple imputation on the findings should be considered when interpreting the results. Conducting such analyses in future studies will enhance the robustness and transparency of the statistical approach. To strengthen the validity of our findings, future research should include multi-center studies with larger sample sizes, standardized assessment timing, and comprehensive data on post-discharge rehabilitation and discharge destinations.

## Conclusions

This study found that IADL abilities were lower in patients transferred to other facilities compared to those discharged home from acute care hospitals. These abilities were significantly influenced by sex, age, days of rehabilitation during hospitalization, and ADL function. While these findings highlight the importance of addressing low ADL capacity in older patients to prevent subsequent IADL decline, further studies are needed to analyze in greater detail the factors contributing to reduced IADL, including the type and intensity of rehabilitation interventions and baseline cognitive and physical conditions. Clarifying these factors will be essential for developing targeted functional and mobility training strategies for patients with cognitive or physical limitations, thereby supporting effective clinical practice.

## Data Availability

The datasets used or analyzed during the current study are available from the corresponding author on reasonable request.
